# Supplementary material for: Sensory perception relies on fitness-maximizing codes
Source: Nat Hum Behav. 2023 Apr 27;7(7):1135–51. doi: 10.1038/s41562-023-01584-y (PMC10365992; doi:10.1038/s41562-023-01584-y)
Supplement: Supplementary file 1 — Supplementary Figs. 1–6, Tables 1–5, and Notes 1 and 2. [file 41562_2023_1584_MOESM1_ESM.pdf]

nature human behaviour

Article https://doi.org/10.1038/s41562-023-01584-y

Sensory perception relies on fitness-maximizing codes

In the format provided by the authors and unedited

Contents

This Supplementary Information document contains

1. Supplementary Figures 1-6
2. Supplementary Tables 1-5
3. Supplementary Notes 1-2

Supplementary Figures

Supplementary Figure 1

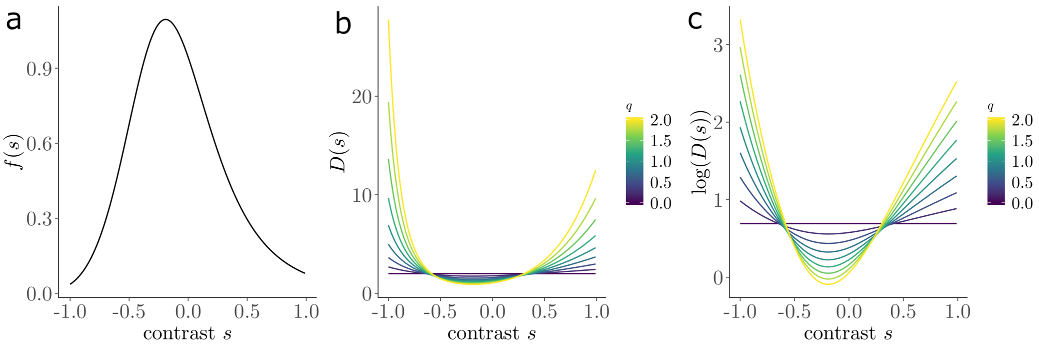

Prior distribution of contrasts in the blowfly and corresponding discriminability thresholds of the power-law efficient codes. a) Prior distribution of contrasts s that the blowfly experiences in its natural environment. Contrasts that appear more often in the natural environment have higher values on the y-axis. In order to estimate the prior distribution, we followed the procedure from previous work (REF. 37 in main text) where the stimulus CDF $F(s)$ was estimated using a 3-parameter Naka-Rushton function

$$F(s) = \frac{|(s-a)^b|}{|s-a|^b + c}.$$

The parameters that best fit the data are $a = -1.52$, $b = 5.80$ and $c = 7.55$ (plotted as the grey dotted line in Fig. 1b). Thus, the prior distribution $f(s)$ shown in this figure was recovered by integrating over $F(s)$.

b, c) Discriminability thresholds as a function of s and power-law value, q. Lower values on the y-axis indicate higher discrimination sensitivity. In order to compute the discriminability thresholds $D(s)$, we used the relationship between $D(s)$ and Fisher information $J(s)$ derived in previous work (REF. 67 in main text). Briefly, assuming that the distribution of stimulus estimations $p(\hat{s} \mid s)$ is normally distributed, it is possible to show that the relationship between $D(s)$ and $J(s)$ obeys

$$D(s) \propto \frac{1}{\sqrt{J(s)}}.$$

According to the power-law efficient codes, the following relationship between $J(s)$ and $f(s)$ holds (see Eq. 9 in Methods)

$$J(s) \propto f(s)^q,$$

thus,

$$D(s) \propto \frac{1}{\sqrt{f(s)^q}}.$$

We use this result to generate discriminability threshold predictions as a function of different power q values and contrast levels s (panel b). Recall that $q = 2$ corresponds to

the Infomax solution, and $q = 4/3$ to the reward-maximization solution (see Methods). These plots clearly show that higher power q values emphasize discriminability for stimuli with higher likelihood of appearing, whereas lower q values lead to greater sensitivity for less common stimuli. Panel c shows $\log(D(s))$ to better visualize the differences in discriminability thresholds.

Supplementary Figure 2

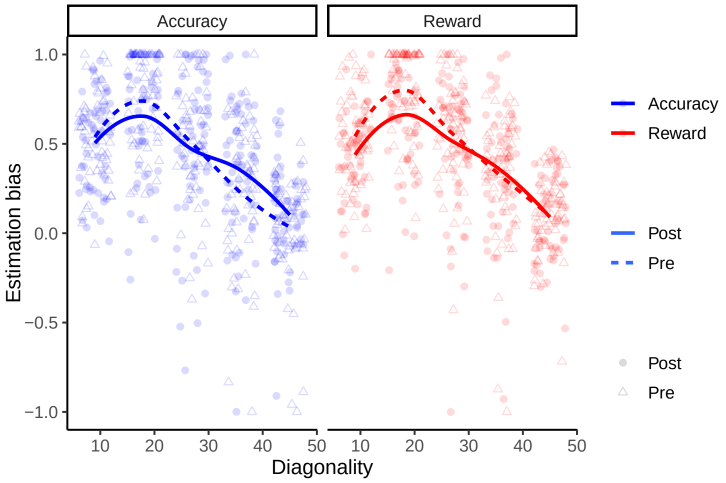

Changes in estimation bias. The dots and lines show the estimation bias before (dotted line, open triangles) and after training (solid line, filled circles) in K_{Rew} (red) and K_{Acc} (blue). Each dot represents the mean bias (reported minus target angle) for a given participant in each of the five diagonality bins. There was a significant decrease in estimation bias following training in both contexts ($P_{MCMC} = 0.02$).

Supplementary Figure 3

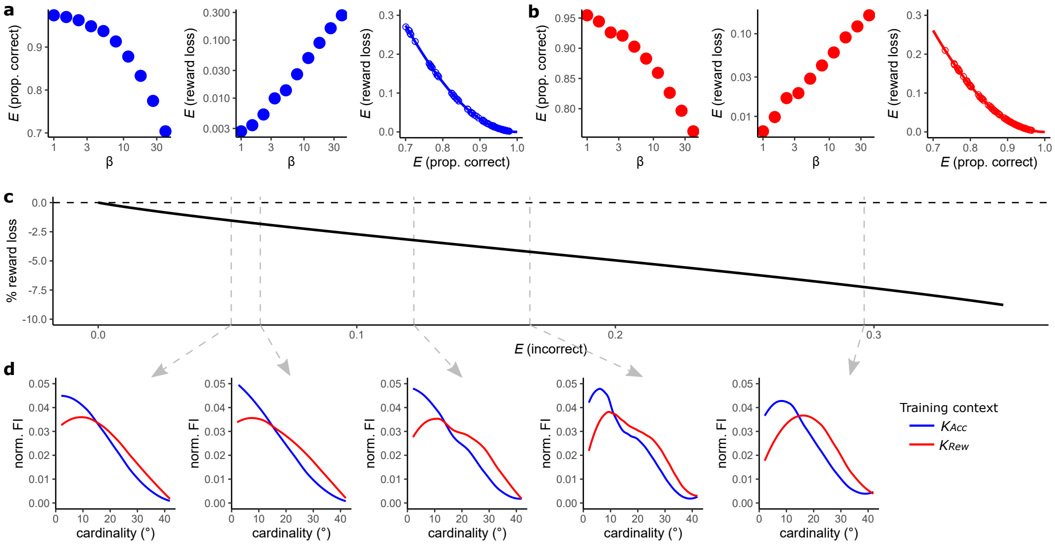

Detailed analyses of the VIB-like objective in the ANNs. a) Performance of the ANN trained in context K_{Acc} in terms of proportion of correct responses (left panel) and expected reward loss (middle panel) as a function of different β values. The relation between the expected reward loss and the expected proportion of correct responses is shown in the right panel for several ANNs trained at different β values (each network is an open circle).

b) Same as panel a but for the ANN trained in context K_{Rew}. The relation between expected reward loss and the proportion of correct responses can be thought of as a rate-distortion curve. We studied the relation of the form:

$$A \cdot (1 - \exp\{b \cdot \log(2E[\text{correct}] - 1)\})^c,$$

where A is the expected reward loss when the expected proportion of correct responses is at chance level (i.e., $E[\text{correct}] = 0.5$). Thus, A is determined by the reward contingencies of the environment (i.e., is not a free parameter), and therefore the free parameters that determine the shape of the curve are b and c. Note that this is a monotonically decreasing function where the extreme points are interpretable: $E[\text{reward loss}; E[\text{correct}] = 0.5] = A$ and $E[\text{reward loss}; E[\text{correct}] = 1] = 0$ as desired. The fits of this curve to the behaviour of the ANNs are the solid lines shown in the right plots of panels a and b, which we found to capture the behaviour of the ANNs very well.

c) We took advantage of the excellent qualitative fits of the relation between the expected reward loss and the expected proportion of correct responses to estimate the proportion of reward loss of the ANN network trained in context K_{Acc} vs the same metric for the ANN trained in context K_{Rew} as a function of the expected proportion of incorrect responses (solid black line). We found that for the range of β values studied here, the ANN trained in context K_{Acc} always led to a reward loss relative to the ANN trained in context K_{Rew}.

d) Fisher's Information (FI) of the second retinotopic layer of the ANN trained in K_{Acc} (blue) and K_{Rew} (red) contexts at different matched levels of accuracy performance across the ANNs (grey dashed arrows in panel c). In line with the analytical predictions, FI is lower for more cardinal angles but higher for more diagonal angles when the network is trained in context K_{Rew} across different levels of ANN accuracy performance (and therefore across different values of β).

Supplementary Figure 4

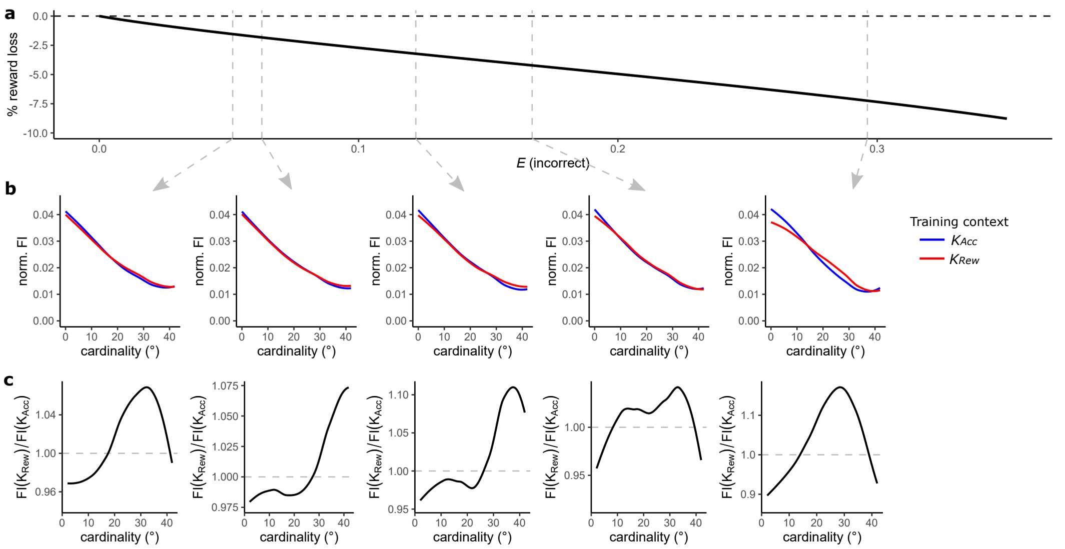

FI at the first retinotopic layer when informational bottleneck pressures are applied in the second layer. a) This panel is reproduced from Figure 2c for convenience. We took advantage of the excellent qualitative fits of the relation between the expected reward loss and the expected proportion of correct responses to estimate the proportion of reward loss of the ANN network trained in context K_{Acc} vs the same metric for the ANN trained in context K_{Rew} as a function of the expected proportion of incorrect responses. We found that for all the space of β values studied here, the ANN trained in context K_{Acc} always led to a reward loss relative to the ANN trained in context K_{Rew}.

b) Fisher's Information (FI) of the first retinotopic layer of the ANN trained in K_{Acc} (blue) and K_{Rew} (red) contexts at different matched levels of accuracy performance across the ANNs (grey dashed arrows in panel a). FI is in general lower for more cardinal angles but higher for more diagonal angles when the network is trained in context K_{Rew} across different levels of ANN accuracy performance (and therefore across different values of β). This pattern is less pronounced relative to the one found in the second retinotopic layer (see Supplementary Figure 2), but it was consistent across different levels of ANN performance as it is evident from the FI ratio between the networks trained in contexts K_{Acc} and K_{Rew} (see panel **c**).

Supplementary Figure 5

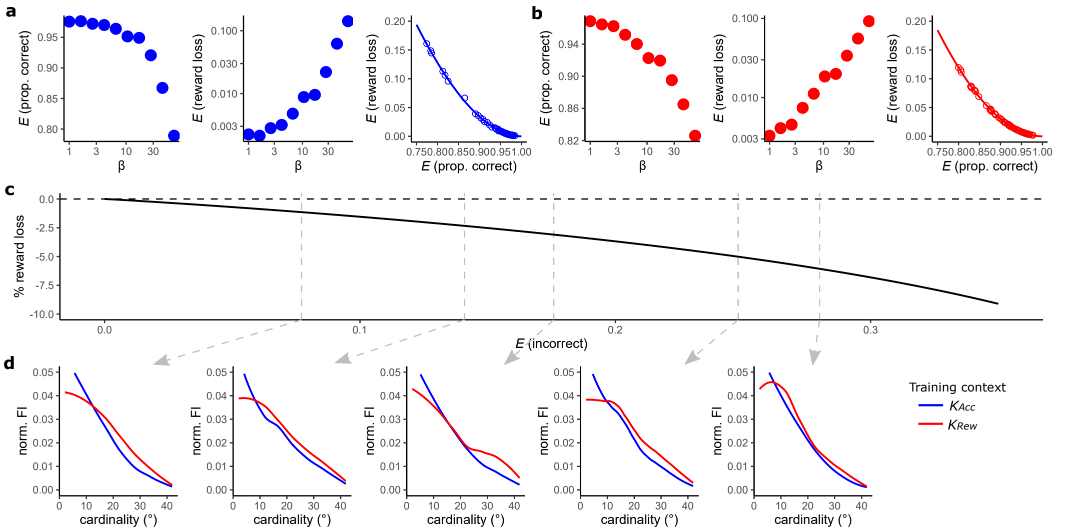

Analyses of the VIB-like objective in the ANNs when informational bottleneck pressures are applied at the decision layer. The approach of this figure is the same as that of Figure Supplementary Figure 3, with the difference that here the informational bottleneck pressure indicated by β was induced at the decision layer. The Fisher's information (FI) analyses presented in panel d are conducted at the second retinotopic layer.

Supplementary Figure 6

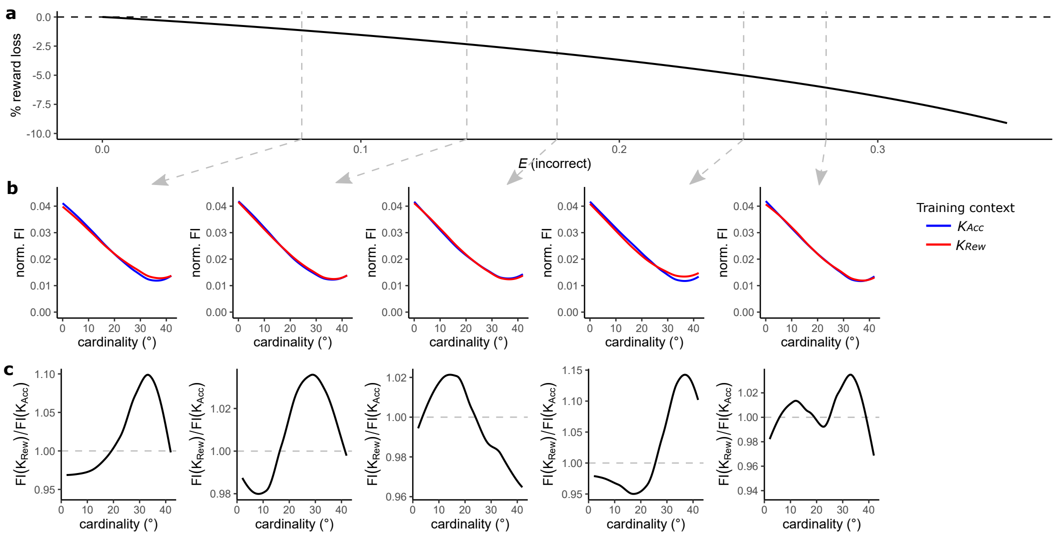

FI at the first retinotopic layer when informational bottleneck pressures are applied in the decision layer. The approach of this figure is the same as that of Figure Supplementary Figure 4, with the difference that here the informational bottleneck pressure indicated by β was induced at the decision layer. The Fisher's information (FI) analyses presented in panels b and c are conducted at the first retinotopic layer.

Supplementary Tables

Table S1. Discrimination accuracy by training context.

	Estimate	Est.Error	Pmcmc
Intercept	76.68	0.54	0.0001
s	-4.50	0.45	0.0001
Krew	-0.14	0.56	0.4067
Later	1.15	0.65	0.0365
*s**Krew	-1.00	0.51	0.0245
*s**Later	0.77	0.51	0.0657
Krew*Later	0.50	0.70	0.2458
*s**Krew*Later	1.46	0.65	0.0118

Tabell S1: *

This table reports the results of a Bayesian hierarchical linear regression on choice performance in the decision task. The regressor Krew is a dummy variable for training type (1 = reward, 0 = accuracy). The regressor *s* is the diagonality of the stimuli. The regressor Later is a dummy variable for later trials in the decision task (1 = later trials, 0 = early trials). The significant interaction *s**Krew*Later indicates that compared to the accuracy context, the reward context led to improved performance for more diagonal stimuli over the course of the task.

Table S2. Response time by training context.

	Estimate	Est.Error	Pmcmc
Intercept	0.69	0.02	0.0001
s	0.04	0.01	0.0001
Krew	0.01	0.01	0.1465
Later	-0.11	0.02	0.0001
*s**Krew	0.01	0.01	0.1548
*s**Later	0.00	0.01	0.4173
Krew*Later	0.01	0.02	0.1827
*s**Krew*Later	0.01	0.01	0.2155

Tabell S2: *

This table reports the results of a Bayesian hierarchical linear regression on response times in the decision task. The regressor Krew is a dummy variable for training type (1 = reward, 0 = accuracy). The regressor s is the diagonality of the stimuli. The regressor Later is a dummy variable for later trials in the decision task (1 = later trials, 0 = early trials). There are no significant main effects or interactions with training context.

Table S3. Changes in the variance in estimation accuracy following reward compared to accuracy training.

	Estimate	Est.Error	Pmcmc
Intercept	-0.09	0.05	0.0317
Krew	0.12	0.06	0.0264
Oblique	0.09	0.07	0.0837
Krew*Oblique	-0.16	0.09	0.0405

Tabell S3: *

This table reports the results of a Bayesian hierarchical linear regression on the variance in estimation accuracy after training in the reward condition (K_{Rew}) versus accuracy (K_{Acc}) conditions. The coefficient Krew indicates that the estimation variance for cardinal orientations increases more following K_{Rew} than K_{Acc} training. The interaction coefficient Krew*Oblique indicates that the estimation variance for oblique orientations decreases more following K_{Rew} than K_{Acc} training.

Table S4. Changes in the variance in estimation accuracy following location-specific reward training.

	Estimate	Est.Error	Pmcmc
Intercept	-0.01	0.05	0.3840
Krew	0.10	0.05	0.0270
Oblique	0.03	0.05	0.2758
Krew*Oblique	-0.12	0.07	0.0433
Response time	0.25	0.25	0.1522
Error magnitude	1.32	0.11	0.0001

Tabell S4: *

This table reports the results of a Bayesian hierarchical linear regression on the estimation variance in the untrained and trained locations for the reward condition. The coefficient Krew indicates that the estimation variance for cardinal orientations increases more in the trained than untrained locations. The interaction coefficient Krew*Oblique indicates that the estimation variance for oblique orientations decreases more in the trained than untrained locations. The regressors for response time and error magnitude control for the simple effects of task experience.

Table S5. Changes in the variance in estimation accuracy following location-specific accuracy training.

	Estimate	Est.Error	Pmcmc
Intercept	-0.00	0.04	0.4740
Kacc	-0.01	0.05	0.3980
Oblique	-0.07	0.05	0.0857
Kacc*Oblique	0.08	0.07	0.1408
Response time	-0.26	0.17	0.0615
Error magnitude	1.42	0.08	0.0001

Tabell S5: *

This table reports the results of a Bayesian hierarchical linear regression on the estimation variance in the untrained and trained locations for the accuracy condition. Estimation variance does not differ between the trained and untrained locations for either cardinal or oblique orientations. The regressors for response time and error magnitude control for the potential effects of location-specific experience.

Supplementary Notes

Supplementary Note 1. Accuracy maximization is not equivalent to fitness-maximization in systems with noisy sensory encoding

The fitness maximization problem that we study here is one in which resource constraints require a compressed encoding scheme with an inherent loss of information. This requirement for compression is present in most sensory systems because the world is too complex to encode in its entirety. According to informational theoretic principles, if information is lost in the encoding-decoding pipeline, it cannot be recovered at later stages of processing irrespective of how complex downstream circuits are. Thus we study the possibility that if early sensory neurons lose specific types of information because they are rigidly fixed to the infomax solution, which favors some bits of information over others, then this information might not be fully available to achieve behavioural goals that require a different compressed representation of features in the environment to optimize behaviour. The neural network analyses described in the main text already provide evidence in favor of this claim. Nonetheless, here we also prove that an infomax solution is not always compatible with fitness-maximizing solutions for the contexts that we consider in our study, and that infomax and fitness-max schemes result in a different informational structure.

To see this, we start by deriving a general fitness-maximizing solution for the kind of reward contingencies that we consider in context K_{Rew} in which each stimulus in the environment is associated with a level of reward (which, as we mentioned in the main text, has ecological validity in economic settings). Here, the goal is to maximize the expected value which is defined in general as $E \equiv Ps$ where P is the probability of obtaining a reward associated to stimulus s. In our problem, this means that we must maximize

$$\mathrm{E}[s(\text{chosen})] = \int\int f(s_1,s_2)\,[P_1(s_1,s_2)s_1 + P_2(s_1,s_2)s_2]\,ds_1 ds_2 \tag{1}$$

where $P_i(s_1,s_2)$ is the probability of choosing option i.

We begin by noting that for any pair of input values s_1, s_2, the integrand in (1) can be written as

$$\begin{aligned}
&P_1(s_1,s_2)s_1 + P_2(s_1,s_2)s_2 \qquad (2)\\
&\quad= \max(s_1,s_2) - P_1(s_1,s_2)\max(s_2-s_1,0) - P_2(s_1,s_2)\max(s_1-s_2,0)\\
&\quad= \max(s_1,s_2) - [P_1(s_1,s_2)I(s_2>s_1) + P_2(s_1,s_2)I(s_1>s_2)]\,|s_1-s_2|\\
&\quad= \max(s_1,s_2) - [P(\text{error}\,|s_1,s_2)I(s_2>s_1) + P(\text{error}\,|s_1,s_2)I(s_1>s_2)]\,|s_1-s_2|\\
&\quad= \max(s_1,s_2) - P(\text{error}\,|s_1,s_2)\,|s_1-s_2|,
\end{aligned}$$

where $I(A)$ is the indicator function (taking the value 1 if statement A is true, and the value 0 otherwise), and $P(\text{error}\,|s_1,s_2)$ is the probability of choosing the lower-valued of the two options.

Substituting this last expression for the integrand in (1), we see that we can equivalently write

$$\mathrm{E}[v(\text{chosen})] \;=\; \mathrm{E}[\max(s_1,s_2)] - \int\!\!\int f(s_1,s_2)\,P(\text{error}\,|s_1,s_2)\,|s_1-s_2|\,ds_1ds_2, \quad (3)$$

where

$$\mathrm{E}[\max(s_1,s_2)] \;\equiv\; \int\!\!\int f(s_1,s_2)\,\max(s_1,s_2)\,ds_1ds_2 \quad (4)$$

is a quantity the value of which is independent of $P(\text{error})$. Hence maximizing the expression above is equivalent to minimize

$$\mathrm{E}[\text{loss}] \;=\; \int\!\!\int f(s_1,s_2)\,P(\text{error}\,|s_1,s_2)\,|s_1-s_2|\,ds_1ds_2. \quad (5)$$

This reduced expression of our optimization problem has an intuitive interpretation: In our reward-maximizing context, given two stimuli s_1 and s_2, the observer is guaranteed to obtain at least the smaller of the two rewards, thus if she chooses the wrong alternative, the reward loss is the absolute difference of the two $|s_1-s_2|$. Thus the problem is solved by computing the expected reward loss weighted by the probability that an error occurs by integrating over all possible combinations of the alternative values.

The solution to this optimization problem requires an assumption about the shape of the distribution of the estimates $\hat{s}_i$. Approximating the Bayesian-mean estimator by a Gaussian distribution with mean value v_i and variance σ_i^2, the difference between the two estimates is normally distributed (c.f., Equation 21 in Methods)

$$g = \frac{v_2 - v_1}{\sqrt{\sigma_2^2 + \sigma_1^2}}. \quad (6)$$

Using the Cramer-Rao bound formulation to approximate the variance, the distribution of the difference between the estimates is given by

$$g = \frac{v_2 - v_1}{\sqrt{\frac{1}{J(v_2)} + \frac{1}{J(v_1)}}}, \quad (7)$$

where J is the Fisher information. Based on this definition, we proceed to minimize the expression in Eq. 5. First, we define Φ as the standard normal CDF. Then the probability of making an erroneous decision is given by

$$P(\text{ error } \mid v_1,v_2) = \Phi(-|g|). \quad (8)$$

Based on this definition, fixing v_1 and integrating over v_2 in Eq. 5 results in approximately

$$\begin{aligned} &\int \Phi(-|g|)|g| \left(\frac{1}{J(v_2)} + \frac{1}{J(v_2)}\right) f(v_2)\,dz \\ \approx &\frac{f(v_1)}{J(v_1)} \int 2\Phi(-|z|)|z|dz, \end{aligned} \quad (9)$$

where following our notation in the main text, $f(s)$ is defined as the prior distribution over the stimulus space s. Note that the integral in the second line is a constant. Therefore, the optimization problem reduces to minimize

$$\int \frac{f(v)}{J(v)} dv. \tag{10}$$

Recall that the problem of minimizing the power-law efficient codes can be written in general as (see Eqs. 9 and 10 in Methods)

$$\underset{J(s)}{\text{argmax}} - \int ds f(s) J(s)^{-\alpha} \tag{11}$$

subject to a capacity bound

$$\int ds J(s)^{\beta} \leq c, \tag{12}$$

and that the solution of this problem is given by

$$J_{\text{opt}}(s) = c^{1/\beta} \left(\frac{f(s)^{\gamma}}{\int ds f(s)^{\gamma}} \right)^{1/\beta}. \tag{13}$$

Note that the expressions in Eqs. 10 and 11 are equivalent if we define $\alpha = 1$. Thus fixing $\beta = 0.5$ and using the solution in Eq. 13 gives

$$J_{\text{opt}}(s) \propto \left(\frac{f(s)^{2/3}}{\int ds f(s)^{2/3}} \right)^{2}. \tag{14}$$

We can proceed in a similar manner by finding the solution that minimizes the number of errors (i.e., that maximizes discrimination accuracy). Minimizing average errors is equivalent to minimizing

$$\int \int f(s_1, s_2) P(\text{error} | s_1, s_2) ds_1 ds_2. \tag{15}$$

Using the same assumptions and similar steps as developed above, we obtain that the optimization problem is reduced to

$$\int \frac{f^2(v)}{J^{1/2}(v)} dv. \tag{16}$$

Substituting the corresponding values of α and γ shows that the solution to the accuracy maximization problem is given by

$$J_{\text{opt}}(s) \propto \left(\frac{f(s)}{\int ds f(s)} \right)^{2}, \tag{17}$$

which is equivalent to the infomax solution $J_{\text{opt}}(s) \propto f(s)^2$.

Thus, the solutions obtained in Eqs. 17 and 14 prove that accuracy maximization is not equivalent to fitness-maximization in the reward contingencies of context K_{Rew} in the discrimination task faced by our participants in Experiments 1 and 2. Also, the fact that Fisher information $J(s)$ is different in both problems suggests that in fine discrimination tasks relying on low level sensory features, it is essential that information that is relevant to maximize reward consumption is already present at the early stages of sensory processing to avoid losing information that by definition cannot be regained in noisy communication systems, such as the brain.

Supplementary Note 2. Efficient representation of sensory information about abstract goal-directed behavioural rules in human early sensory areas

Our hypotheses and empirical tests are based on the premise that, in most real-life situations, the value of an action is strongly tied to the behavioural goal that an agent seeks to achieve. To further test this hypothesis, we re-analyzed data from a recent human fMRI study to investigate whether novel goal-directed actions that promote an individual's survival"in hypothetical scenarios they've never before encountered trigger efficient reorganization of perceptual information (REF. 55 in main text).

In this experiment, human volunteers underwent fMRI while they imagined using different visually presented items to achieve two distinct goals. In short, participants were asked to picture themselves as pilots of a cargo aircraft flying over the ocean at night. A sudden engine failure required an emergency landing on a deserted island. The final objective of the task was to flee the island: To do so, participants could use each of the items retrieved from the aircraft wreck (Figure SN2a). Two possible escape strategies are proposed to the participants, each requiring the achievement of a separate goal: either starting a fire to be detected by a rescue team (hereafter referred to as burning goal) or keeping a boat anchored ashore until setting sail in the morning (hereafter referred to as anchoring goal, see Figure SN2d).

To test the effect of goal manipulations on brain activity, Castegnetti and colleagues used representational similarity analyses (RSA) in a whole-brain volumetric searchlight approach. RSA is a multivariate technique that is well-suited for the study of the representational content of brain activity patterns. By assuming efficient compression of information in an information bottleneck system, RSAs allow one to elucidate how well specific brain structures can disentangle information in an efficient manner. Notably, to build these representations, participants had to consider the consequences of their actions rather than applying fixed stimulus-action maps.

The first hypothesis proposed by Castegnetti and colleagues was that visual areas support goal-independent item representations that are determined by the item's perceptual features, but are insensitive to behavioural goals. Their second hypothesis proposed that downstream, higher-level association areas, such as the vmPFC, would represent items in a novel abstract space that integrates item and goal, such as the degree of usefulness of the item.

However, our hypothesis about representations in visual and object recognition areas was qualitatively different than the original authors' hypotheses. We predicted that, due to an information bottleneck in the visual system, efficient representation of low-level sensory features that are relevant for the task at hand must occur as early as possible in order to encode and disentangle information before it is lost. Note that this does not preclude sensory representations of an object's identity as well as its goal relevance. Thus, we hypothesized that object-representation structures such as LOC (a brain structure known to be involved in disentangling information for object recognition) and possibly also visual areas such as

V1-V3 should be involved in representing behaviourally relevant sensory information, as opposed to only in downstream regions.

In general the data were inline with both the original authors' hypotheses and our own. The RSA analyses reveal that activity patterns consistent with object identity representations are present in a single large cluster encompassing portions of the occipital lobe and extending into the temporal lobe, including primary visual areas and the LOC. Patterns representing sensory information in the service of novel goal-directed behaviour (henceforth referred to *usefulness* of sensory information) were found in prefrontal structures such as the vmPFC and the OFC (Figure SN2d). However, and critically for our hypothesis, when examining the same RSA maps generated by Castegnetti and colleagues, we found that the usefulness of objects was not just represented in downstream regions, but was already present in LOC and early visual areas such as V2 and V3 (Figure SN2d-g).

These human brain imaging analyses reveal that efficient representation of sensory information about context-specific, goal-directed behavioural rules that aim at maximizing reward (or hypothetical survival of the participant as it was framed in the behavioural task) also occur at early stages of cortical sensory processing. This indicates that our conclusions hold not only for insect retina but also for human visual cortex.

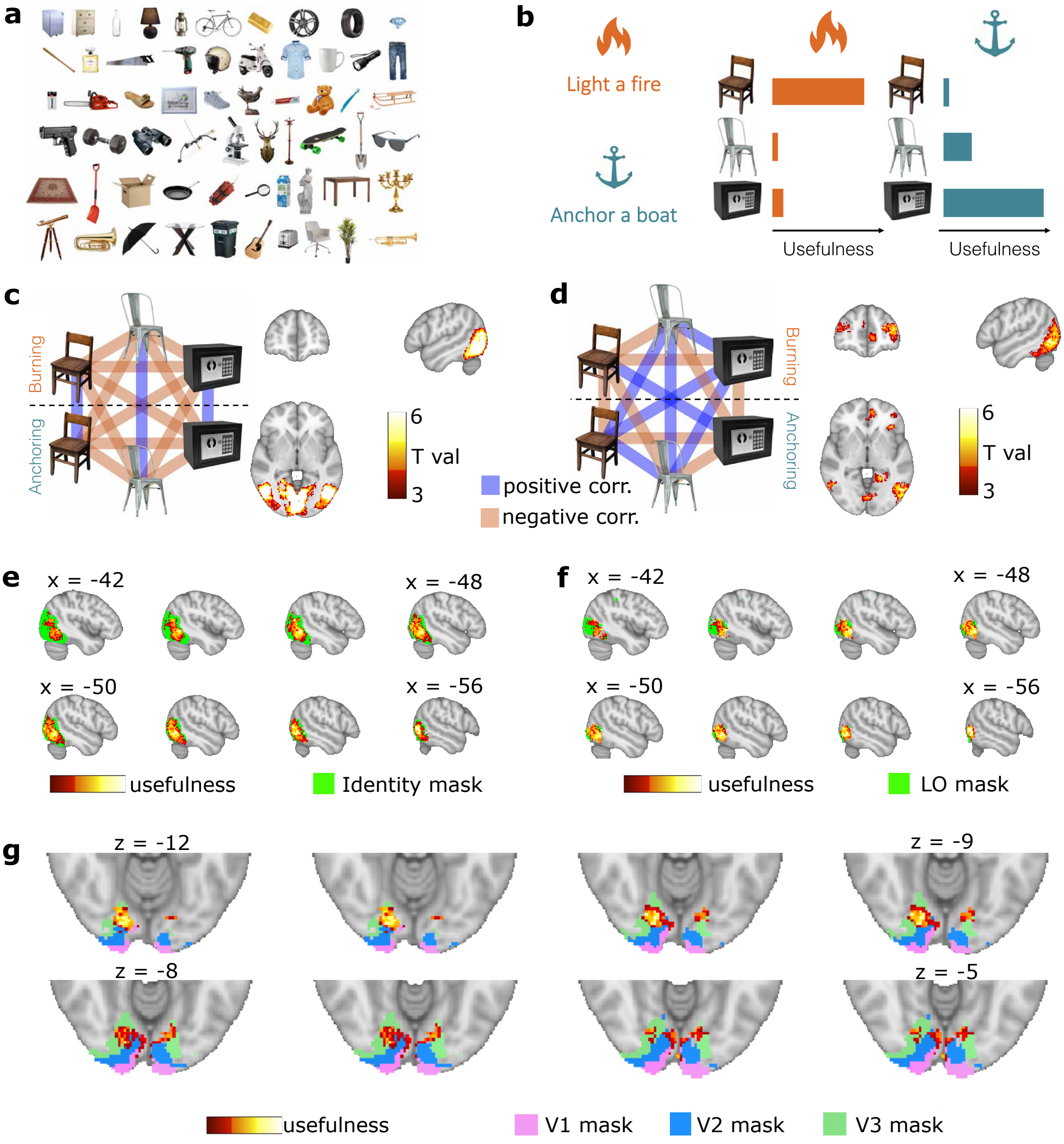

Figure SN2. Novel goal-directed utilities that rely on low level sensory features are efficiently represented at early stages of sensory processing. a) A sample of the objects used in the task. **b)** On day 1, participants evaluated the usefulness of each object for each goal (anchoring or burning) in the hypothetical survivalscenario described in the behavioural task. Note that an item's usefulness depended on the decision-maker's goal. For instance, Castegnetti and colleagues give the example of goal-specific usefulness values for a wooden chair that is useful for burning, unlike a perceptually similar metal chair or a heavy safe. In contrast, the safe and, to some extent, the metal chair, might be useful for anchoring, while the wooden chair is not useful for this goal because it floats. On the second day of the experiment, fMRI data were acquired while participants viewed the objects from day 1. During the fMRI scans, participants were asked to imagine using the current item for a given goal-directed action (anchoring or burning). **c,d)** (Left) Schematic depictions of the representations of item identity (c) and usefulness (d). Blue and red lines indicate positive and negative correlations, respectively.

(Right) Brain regions whose activity follows the sensory features under both goals (i.e., object identity, c) and the novel goal-directed representation (usefulness of the object for survival strategy, d), based on RSA searchlight analysis. All statistical maps (heat colourbar) show voxels surviving FWE-correction ($P < 0.05$) at the cluster level. The FWE corrections were computed using a cluster-defining threshold of $P < 0.001$ and permutation tests. As expected, object identity representations are present in early sensory areas including V1-V3 and LO (Identity mask in panel e). The novel and context-dependent goal-directed representations of usefulness are present in vmPFC and OFC, as originally reported (REF. 55 in main text), but we show that they are also present in early visual areas. **e)** Conjunction analysis between statistical maps of object identity and the context-dependent representations of usefulness. This conjunction analysis shows a high degree of overlap for both representations in LOC. **f)** Same as (e), but this time the reference mask (LOC mask) was obtained from the fMRI meta-analysis tool Neurosynth with the keyword Lateral Occipital Cortexänd thresholded at the Neurosynth default of $P < 0.01$ FDR-corrected. **g)** V1-V3 masks were extracted from the Julich-Brain Cytoarchitectonic Atlas and thresholded at 50% probability. The V1-V3 masks were then conjoined with the cluster-corrected statistical map of usefulness representations. Goal-dependent representations of usefulness are found in early visual cortex (primarily V2-V3), suggesting that low-level sensory details determining an object's goal-specific usefulness are also represented and distinguished at early stages of sensory processing. Objects depicted in panels a-d were adapted from REF. 55 (main text), work distributed under a Creative Commons Attribution License 4.0 (CC BY).
